# Long Non-Coding RNA FGD5-AS1 Induced by *Chlamydia trachomatis* Infection Inhibits Apoptosis *via* Wnt/β-Catenin Signaling Pathway

**DOI:** 10.3389/fcimb.2021.701352

**Published:** 2021-09-09

**Authors:** Yating Wen, Fangzhen Luo, Lanhua Zhao, Shengmei Su, Wenbo Lei, Yi Liu, Keliang Shi, Zhongyu Li

**Affiliations:** Institute of Pathogenic Biology, Hengyang Medical College, Hunan Provincial Key Laboratory for Special Pathogens Prevention and Control, Hunan Province Cooperative Innovation Center for Molecular Target New Drug Study, University of South China, Hengyang, China

**Keywords:** *Chlamydia trachomatis*, long non-coding RNA, apoptosis, Wnt/β-catenin signaling pathway, FGD5-AS1

## Abstract

**Background:**

*Chlamydia trachomatis* (Ct) is one of the most common bacterial sexually transmitted infection (STI) pathogens in the world, but the exact pathogenic mechanism still needs to be further elucidated. Long non-coding RNAs (lncRNAs) have become vital regulators in many biological processes. Their role in the interaction between Ct and host cells has not been reported.

**Methods:**

Microarrays were used to study the expression profiles of lncRNAs and mRNAs in HeLa cells at 12, 24, and 40 h post-infection (hpi). Differentially expressed lncRNAs and mRNAs were verified by RT-qPCR. Coding-non-coding (CNC) network analysis showed co-expression molecules of selected lncRNA. Western blot, flow cytometry, and indirect immunofluorescence were used to detect the effect of lncRNA FGD5-AS1 on apoptosis during Ct infection.

**Results:**

Compared with the uninfected group, the number of differential lncRNAs were 2,130, 1,081, and 1,101 at 12, 24, and 40 hpi, and the number of differential mRNAs was 1,998, 1,129, and 1,330, respectively. Ct induced differential expression of large amounts of lncRNAs and mRNAs in HeLa cells, indicating that lncRNAs may play roles in the pathogenesis of Ct. RT-qPCR verified six differential lncRNAs and six differential mRNAs, confirming the reliability of the microarray. Among these molecules, lncRNA FGD5-AS1 was found to be upregulated at 12 and 24 hpi. Coding-non-coding (CNC) network analysis showed that co-expressed differential molecules of FGD5-AS1 at 12 and 24 hpi were enriched in the DNA replication and Wnt signaling pathway. The downregulation of FGD5-AS1 decreased the expression of β-catenin and inhibited the translocation of β-catenin and the DNA replication, while it promoted apoptosis of the host cells.

**Conclusions:**

DNA replication and apoptosis of host cells were affected by upregulating FGD5-AS1 *via* Wnt/β-catenin pathway during Ct infection. This study provides evidence that lncRNAs are involved in the coaction between Ct and hosts, and provides new insights into the study of lncRNAs that regulate chlamydial infection.

## Introduction

*Chlamydia trachomatis* (Ct) is an obligate intracellular Gram-negative microorganism. It is one of the leading clinical pathogens causing blindness and bacterial sexually transmitted infection (STI). Chlamydial infection of the reproductive tract causes serious complications, such as epididymitis and prostatitis in males, and cervicitis, pelvic inflammatory disease, fallopian tube inflammation, ectopic pregnancy, and infertility in females (WHO Alliance for the Global Elimination of Blinding Trachoma by the year 2020). Ct infection is closely associated with ovarian cancer and is an important risk factor of HPV-induced cervical cancer. However, the exact molecular mechanism of Ct is still unclear.

Ct has a unique development cycle, which is transformed between two different forms. The initiation of developmental cycle is the adhesion and entry of metabolically inactivated infectious elementary bodies (EBs) into the host cell. EBs rapidly differentiate into proliferative but non-infectious reticular bodies (RBs). After replication, RBs differentiate asynchronously into EBs. Re-differentiated EBs leave the host cell by lysis or extrusion to infect neighboring cells (Chen et al., 2019).

In order to maintain a stable subcellular environment to complete the development cycle, Ct develops a variety of mechanisms to interact with host cells, such as interfering with autophagy and escaping host cell immune response (Murray and McKay, 2021). It has been shown that Ct can influence host transcriptome and proteome profiles for regulating cell signaling pathways and inhibiting host cell death (Hayward et al., 2019). As one of the key strategies for intracellular survival, the regulation of Ct infection on apoptosis is intricate. Ct has obviously timeliness in regulating apoptosis: the host cell death is inhibited in the early stage of infection to protect the survival of host cells, while it is induced to promote EB spread in the late stage. If the host cell apoptosis is induced early, it will repress Ct propagation, and the mechanism of its resistance to apoptosis has not been fully elucidated.

The interaction between Ct and host is achieved by affecting the expression of key molecules in biological processes, and protein expression is usually regulated by non-coding RNAs (ncRNAs) including long non-coding RNAs (lncRNAs) (Wen et al., 2020a; Zhu et al., 2021). LncRNAs are the kind of ncRNAs with a length of more than 200 nucleotides, which regulate gene expression at multiple levels *via* various mechanisms, thus influencing development, differentiation, and metabolism (Batista and Chang, 2013; Fatica and Bozzoni, 2014). In recent years, the research on lncRNAs in bacterial infection have focused on intracellular microorganisms. For example, lncRNA MIR3954 HG-V1 and V2 were upregulated after *Mycobacterium tuberculosis* (Mtb) infection, which can be regarded as the candidate diagnostic markers for Mtb infection (Yang et al., 2016). *Helicobacter pylori* (Hp) activated SGK1/JunB signaling pathway by upregulating lnc-SGK1, inhibiting Th1 cell differentiation and promoting Hp infection and proliferation (Yao et al., 2016). Previous studies showed that the secreted plasmid protein pORF5 of Ct improved the survival of host cells by upregulating ZFAS1 via activation of MAPK/p38 pathway (Wen et al., 2020b). However, we still know little about the role of lncRNAs on the signaling pathway in response to the obligate intracellular bacterium during Ct pathogenesis.

**Figure 1:**
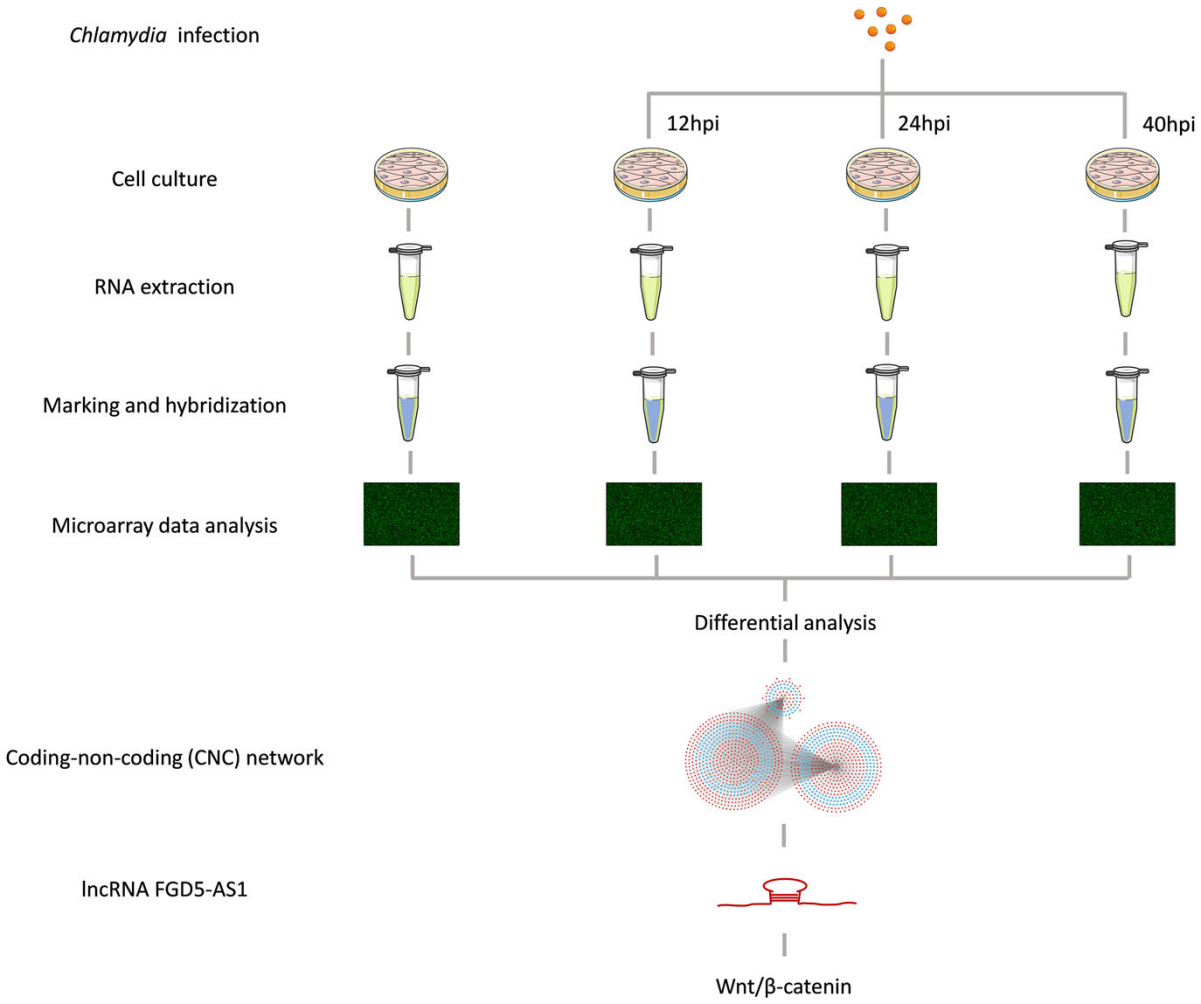
The schematic flow of the experiment. Part of elements come from SMART (https://smart.servier.com).

In this study, we constructed a Ct infection model to analyze the role of host lncRNAs during Ct infection. Based on the previous studies, Ct showed a 16 h incubation period and then proliferated rapidly and reached its peak at 40 h (Belland et al., 2003). Therefore, we selected 12, 24, and 40 h to dynamically monitor the expression of host lncRNAs and mRNAs during the growth cycle of Ct. The flow of this experiment is shown in the **Figure 1**. At 12, 24, and 40 h, the number of host differential lncRNA was 2,130, 1,081, and 1,101, while the differentially expressed mRNA number was 1,998, 1,129, and 1,330, respectively. Ct induced the differential expression of lncRNAs and mRNAs in HeLa cells, among which the number of differential molecules was the most at 12 h. This is consistent with the state that Ct infection affects host physiological process and prepares for intracellular proliferation in the early stage (Elwell et al., 2016).

It is worth noting that both previous research and our experimental results demonstrated that Ct could resist host cell apoptosis at 12 and 24 h, suggesting that apoptosis-related signaling pathways in these two time points were regulated by Ct. The expression of the differential lncRNA FYVE, RhoGEF, and PH domain containing five antisense RNA 1 (FGD5-AS1) was found to be upregulated at both two time points. The co-expressed differential mRNAs were enriched in DNA replication and Wnt signaling pathway. The downregulation of FGD5-AS1 reduced the expression of β-catenin, promoted the apoptosis, and inhibited the translocation of β-catenin and DNA replication of Ct-infected cells at 12 hpi. Our study has shown that Wnt/β-catenin pathway was activated by up-regulating FGD5-AS1 when Ct infection, which influenced DNA replication and apoptosis of host cells. This study provides an important basis for lncRNAs to participate in the interaction between Ct and host and provides a new insight for the study of the pathogenesis of Ct and screening of potential intervention targets.

## Materials and Methods

### Cell Culture and Chlamydia Infection

The human cervical cancer cell line HeLa was cultured in Dulbecco’s Modified Eagle Medium (DMEM; Hyclone, Logan, UT, USA) supplemented with 10% fetal bovine serum (FBS; Gibco, Waltham, MA, USA). Cells were grown at 37°C, 5% CO_2_. When the cells grew to fusion of 90% in a culture flask, they were digested with trypsin (Hyclone) and transferred to a six-well plate and incubated overnight. The cells infected with Chlamydial organisms of Ct serovar E at a multiplicity of infection (MOI) of 0.5 diluted in DMEM supplemented with 10% FBS for 20 min after pretreated with DEAE-dextran (45 µg/ml) for 10 min. Then plates were centrifuged 60 min at 900 × g. Complete culture medium supplemented with 10% FBS, 1 µg/ml cycloheximide (Abcam, Cambridge), and 10 µg/ml gentamycin (Merk, Darmstadt, Germany) were added into infected cells and cultured at 37°C, 5% CO_2_ (Scidmore, 2005; Wen et al., 2020d).

### RNA Extraction

Total RNAs from cells infected with Ct for 12, 24, and 40 hpi and uninfected cells were extracted using TRIzol reagent (Invitrogen, Carlsbad, CA, USA) according to the manufacturer’s protocol. RNA quantification and purity were measured by ND-1000 spectrophotometer (NanoDrop Technologies, Inc., Wilmington, DE, USA). The absorbance ratios of A_260_/A_280_ and A_260_/A_230_ were measured by standard denaturing agarose gel electrophoresis to evaluate RNA integrity.

### Marking and Hybridization

Sample labeling and array hybridization were performed according to the manufacturer’s protocol (Arraystar, Rockville, MD, USA). In short, total RNA was digested with RNase R (Epicentre, Madison, WI, USA) to remove linear RNA. Using an RNA labeling kit (Arraystar, Rockville, MD, USA), each sample was amplified and transcribed into fluorescent cRNA using a random primer method. The labeled cRNA was purified using RNeasy Mini Kit (Qiagen, Hilden, Germany). The concentration and specific activity of the labeled cRNA were measured by a NanoDrop spectrophotometer. One μg of labeled cRNA was lysed by the addition of 1 μl of 25× fragmentation buffer and 5 μl of 10× blocking reagent, then the mixture was heated at 60°C for 30 min, and finally 25 μl of 2×GE hybridization buffer was added to dilute the labeled cRNA. Fifty μl of the hybridization solution was dispensed into spacer slides and assembled on lncRNA expression microarray slides. Slides were incubated in a hybridization oven (Agilent, Santa Clara, CA, USA) at 65°C for 17 h. The hybridized array was washed, fixed, and scanned using Arraystar lncRNA microarray scanner (part number G2505C).

### Microarray Data Analysis

Microarray data collection was performed by Kangchen Biotechnology (Shanghai, China) experimental workflow (GSE165628) (Shi and Shang, 2016). In the Ct-infected cells, lncRNAs with a fold change (FC) of expression ≥2.0 (*P* value <0.05) compared to control cells were selected for further analysis.

### Transmission Electron Microscopy (TEM)

The cells at 12, 24, and 40 hpi were collected by trypsin and fixed by 2.5% glutaraldehyde for 2 h and were centrifuged at 1,200 rpm for 10 min to collect. The supernatant was replaced with 1% osmic acid. Then, samples were dehydrated by using increasing grades of ethanol (50, 70, and 100%) and acetone (90 and 100%) before embedding in a mixture of Spurr epoxide resin (EPON812, DDSA, MNA, and DMP30) and 100% acetone. Ultrathin sections (approximately 70 nm) were stained with uranyl lead citrate and acetate. Finally, inclusions in samples were examined by Tecnai G2 transmission electron microscope.

### RT-qPCR

After treated with DNase without RNase, 2 μg of total RNA in a 20 μl reaction was used for the first-strand synthesis according to the instructions of the SuperScript™ RT-PCR first-strand synthesis system (Invitrogen, Carlsbad, CA, USA). The RT product (1 μl) was used as a template for RT-qPCR in the LightCycle 96 instrument (Roche, Basel, Switzerland) by using SYBR Green I (Tiangen, China). The reaction was performed during 30 cycles of 30 s denaturing at 95°C, 30 s annealing at 60°C, and 30 s extension at 72°C. The *18S rRNA* was used as an internal control. Reactions were performed in triplicate at each time point. The data were normalized to the ratio of lncRNA or mRNA to *18S rRNA* transcript. The relative expression levels were calculated by the delta-delta-Ct method and U-test, respectively. Sequences of the primers used are listed in **Table S1**.

### Bioinformatics Analysis (GO, KEGG, and CNC)

Gene ontology (GO) analysis (www.geneontology.org) was used to study the biological function of differentially expressed coding genes (DGs). The analysis classified functions according to the following three aspects: biological processes (BP), cellular components (CC), and molecular functions (MF). Fisher’s exact test was used to classify GO categories. The *P*-value indicated the importance of GO term enrichment in DGs. The lower the *P*-value, the more meaningful the GO term (*P*-value <0.05).

According to the Kyoto Encyclopedia of Genes and Genomes (KEGG), Biocarta, and Reactome (http://www.genome.jp/kegg/), DGs were used for pathway analysis. The *P*-value (EASE score, Fisher-*P* value, or Hypergeometric-*P* value) indicated the importance of the pathway related to the condition. *P*-value <0.05 was considered statistically significant.

The coding-non-coding co-expression network (CNC) of lncRNA-mRNA was constructed by Co-Lnc software (http://bio-bigdata.hrbmu.edu.cn/Co-LncRNA/). The modified Pearson correlation coefficient (PCC) was used to cut off and to select the lncRNA-mRNA pair, whose PCC ≥0.7. Cytoscape 3.7.1 was used to visualize a CNC network.

### Cell Transfection

To silence lncRNA FGD5-AS1, siRNA targeting lncRNA (si-FGD5-AS1) and NC siRNA were synthesized by RiboBio (Guangzhou, China). The sequence of si-FGD5-AS1 was as follows: 5’-TCACTAAGCTTCACAGATA-3’. Transfection was performed using Lipofectamine 2000 (Invitrogen) according to the manufacturer’s protocol. Twelve hours after transfection, the transfected cells were collected and used for further experiments.

### Western Blot Analysis

Cells were lysed with lysis buffer containing protease and phosphatase inhibitors on ice for 10 min, and then centrifuged (12,000 × g) at 4°C for 10 min. Supernatants were denatured by loading buffer containing 5% β-mercaptoethano, followed by boiling at 100°C for 5 min. SDS-PAGE was performed and transferred to a polyvinylidene fluoride (PVDF) membrane (0.22 μm; Millipore, Bedford, MA, USA) using a semi-dry Trans-Blot SD device (BioRad). At room temperature, the membrane was blocked with blocking buffers (EpiZyme, China) for 15 min. The membrane was incubated with the anti-mouse antibodies to β-catenin and β-actin, and the anti-rabbit antibodies to Bcl-2 and Bax (Danvers, MA, USA), respectively, overnight at 4°C, then goat anti-mouse IgG and goat anti-rabbit IgG (Abcam, Cambridge, UK) conjugated with horseradish peroxidase (HRP) were incubated at 37°C for another 1 h. Protein bands were detected using the enhanced chemiluminescence Western blot system G:BOXChemi XXX9 (Syngene, Cambridge, UK), and analyzed by Quantity One.

### Immunofluorescence Assay (IFA)

Infected HeLa cells grown on coverslips at different time points were fixed with 4% paraformaldehyde in PBS for 30 min, permeabilized with 0.3% (v/v) Triton X-100 in PBS for 10 min, and blocked by DMEM containing 10% FBS for 1 h. The samples were washed with PBS and incubated with specific primary antibodies at 37°C for 2 h. The specific primary anti-mouse antibody to chlamydial reference pORF5 protein was purified and stored as described according to published work (Li et al., 2008). Cy3-labeled goat anti-mouse IgG (Jackson Immuno Research Laboratories, West Grove, PA, USA) and 6-dim-2-phenylindole dihydrochloride (DAPI; Sigma -Aldrich, Munich, Germany) were visualized to β-catenin and nuclear DNA, respectively. After co-incubation for 1 h, the nuclear translocation of β-catenin was observed on a fluorescence microscope (Nikon in Tokyo, Japan).

Hoechst staining was used to detect apoptosis. After the TNF-α was treated for 6 h or other appropriate treatment, the cells were fixed and permeabilized. Then, the cells were incubated with Hoechst 33258 (Beyotime Biotech, Nanjing, China) for 30 min. Apoptotic cells were observed under a fluorescent microscope. The apoptosis rate was determined by the following formula: apoptosis rate = number of apoptotic cells in five arbitrary units/total number of cells in five arbitrary units × 100%.

### Acridine Orange/Ethidium Bromide (AO/EB) Fluorescence Assay

At the appropriate time points, HeLa cells or Ct-infected cells cultured in 24-well plates were added with 10 μl of the dyes AO and EB (Bestbio Science, Shanghai, China). After 10 min, apoptotic cells were observed by using a fluorescence microscope. While red-stained cells suggested that the cell membrane was damaged, which means cell apoptosis, green-stained cells indicated that the cell membrane was intact and alive.

### Flow Cytometry (FCM)

Apoptotic cells were measured using Annexin V-APC/PI apoptosis detection kit (Keygen, China). Briefly, cells were washed with PBS and resuspended in 500 µl of binding buffer, and then 5 µl of Annexin V-APC and 5 µl of propylene iodide (PI) were added. After incubating the cells at room temperature in the dark for 15 min, cells were analyzed by FCM. Cell cycle was detected using cell cycle and apoptosis analysis kit (US EVERBRIGHT^®^ INC., Suzhou, China). Cells were digested with trypsin and washed with PBS. Subsequently, 250 μl of PBS was used to resuspend the cells, and 750 μl of absolute ethanol was used to fix the cells overnight. Before staining, the cells were centrifugated 5 min with 1,000 × g to remove the ethanol and were resuspended by 1 ml of cold PBS. Then 0.5 ml of PI was added to each sample. After incubating the cells at room temperature in the dark for 30 min, the stained cells were analyzed by FCM. FlowJo 7.6.1 software was used to analyze apoptotic rate and cell cycle.

### Statistical Analysis

All results were expressed as the mean ± standard deviation (SD) of three independent experiments. Statistical analysis was performed using *t*-test. All statistical tests were performed using GraphPad Prism 6.0 (GraphPad Software Inc., La Jolla, CA, USA). All statistical tests were indicated by two tails, and *P*<0.05 was considered significant.

## Results

### Establishment of Acute Infection Model of Ct Serovar E

**Figure 2:**
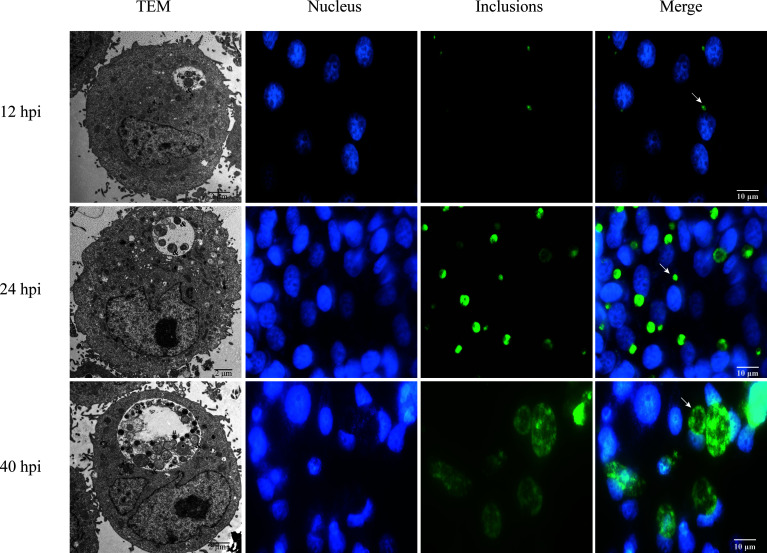
Acute infection of Ct. HeLa cells were infected with Ct at 12, 24, and 40 hpi. The left panel exhibited the inclusions in cells that were mixed and detected by TEM. The symbol of # presents EBs, and & indicates RBs. Other panels exhibited that the infected cells were fixed at different time points. DNA (blue) was stained by Hoechst 33258, and inclusions (green) were stained by Cy2. Arrows denote inclusions.

In order to investigate the alternation of host lncRNA and mRNA induced by Ct, HeLa cells were infected with Ct serovar E organisms for 12, 24, and 40 h (MOI=0.5). To detect the infection of Ct at different time points, IFA and TEM were performed. Results are as shown in **Figure 2**; the inclusion size increased with the infection time. The ultrastructure of inclusions was observed under TEM. At 12 and 40 h, there were dense EB and loose RB, while only RB existed in inclusion at 24 h, which proved that the model of acute Ct infection was successfully constructed.

### Overview of Differentially Expressed lncRNAs and mRNAs

To explore the host lncRNAs associated with Ct infection, we examined the expression profiles of lncRNAs and mRNAs in HeLa cells at different time points by microarray analysis (**Figure S1**). Hierarchical clustering and volcano diagrams confirmed the expression patterns of lncRNAs and mRNAs. The changes in lncRNAs and mRNAs expressions were evaluated by volcano plots (**Figure S2**).

**Figure 3:**
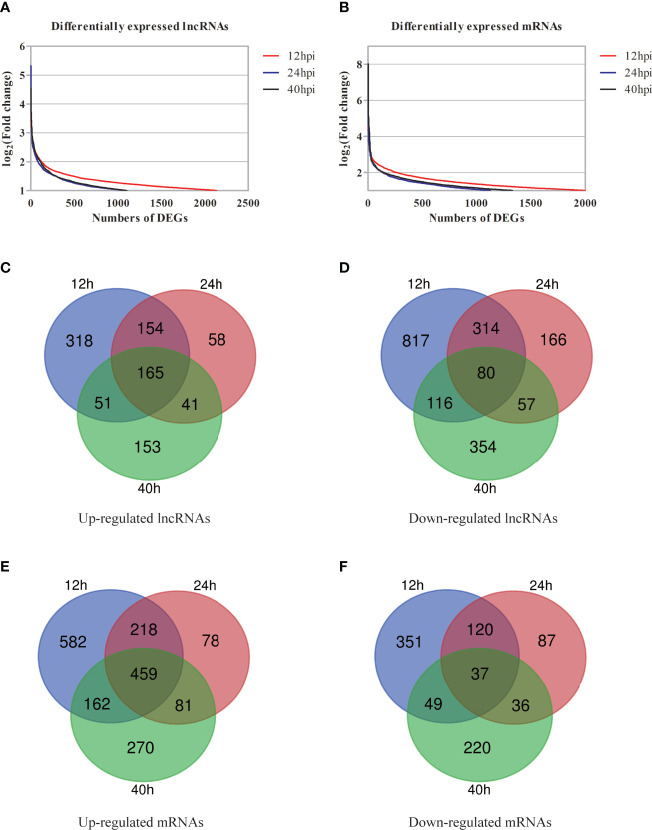
Preliminary overview of DGs at different post-infected hours. **(A, B)** The distribution of differentially expressed lncRNAs **(A)** and differentially expressed mRNAs **(B)** at different post-infected hours shown by scatter plots. **(C, D)** Venn diagrams indicate the numbers of overlapping and non-overlapping upregulated lncRNAs **(C)** and downregulated lncRNAs **(D)**. **(E, F)** Venn diagrams indicate the numbers of overlapping and non-overlapping up-regulated mRNAs **(E)** and downregulated mRNAs **(F)**.

Compared with the uninfected group, 2,130 of lncRNAs (732 of upregulation and 1,398 of downregulation), 1,081 of lncRNAs (442 of upregulation and 639 of downregulation), and 1,101 of lncRNAs (465 of upregulation and 636 of downregulation) were differentially expressed at 12, 24, and 40 hpi, respectively (FC≥2 and *P <*0.05) (**Figures S3A–C**). Compared with the uninfected group, the top 30 of differentially expressed lncRNAs at each time point were listed in supplementary data (**Tables S2**). Venn diagram of differentially expressed lncRNAs showed that 245 of lncRNAs had the same change tendency, including 165 of upregulation and 80 of downregulation (**Figure 3**).

mRNA expression profile data showed that compared with the uninfected group, 1,998 of mRNAs (1441 of upregulation and 557 of downregulation), 1,129 of mRNAs (849 of upregulation and 280 of downregulation), and 1,330 of mRNAs (988 of upregulation and 342 of downregulation) were differentially expressed at 12, 24, and 40 hpi, respectively (FC≥2 and *P*<0.05) (**Figures S3D–F**). The top 30 of differentially expressed mRNAs are showed in **Tables S3**. Venn diagram of the differentially expressed mRNAs showed that there were 496 mRNAs with the same change trend at three time points, of which 459 were upregulated and 37 were downregulated (**Figure 3**).

FC of differentially expressed molecules was transformed with log2(FC)≥1 to analyze the DGs at different time points (**Figures 3A, B**). The results showed that the number of DGs was the largest at 12 hpi. To sum it up, molecular events (such as lncRNAs and mRNAs) in cells have altered after Ct infection.

### GO, KEGG, and Protein-Protein Interaction Network (PPI) Analysis

To further explore the overall effect of Ct infection in HeLa cells, GO analysis was performed on DGs. The results showed that the upregulated differentially expressed mRNAs were mainly enriched in BP related to nucleic acid metabolism, such as “Nucleic acid metabolic process,” CC including “Intracellular organelle lumen,” and “Nucleic acid binding” in MF (**Figures S4A, C, E**). However, the downregulated mRNAs were quite distinct at different time points. The downregulated mRNAs at 12 hpi were mainly enriched in BP related to immunity such as “Innate immune response,” CC including “Membrane part,” as well as “Signal transducer activity” in MF (**Figure S4B**). Downregulated mRNAs at 24 hpi were enriched in BP such as “Terpenoid metabolic process,” CC including “Intrinsic component of membrane,” as well as MF associated with “Signaling receptor activity” and “Molecular transducer activity” (**Figure S4D**). Downregulated mRNAs at 40 hpi were primarily enriched in BP such as “Endocytosis,” and CC including “Cytoskeleton,” as well as MF correlated to “Ligand-gated ion channel activity” (**Figure S4F**). The above results suggest that Ct affects host signal transduction and cell metabolism during infection.

KEGG pathway analysis was used to study the biological pathways involved in differentially expressed mRNAs. KEGG results showed that the upregulated DGs were mostly enriched in “Spliceosome”, “RNA transport”, “Epstein-Barr virus infection”, “Cell cycle”, “NOD-like receptor signaling pathway”, and “DNA replication” and other channels (**Figures S5A, C, E**). However, downregulated DGs were enriched in various signaling pathways at different time points, such as “Calcium signaling pathway” and “cAMP signaling pathway” at 12 hpi; and “Hippo signaling pathway” at 24 and 40 hpi (**Figures S5B, D, F**).

Metascape software analyzed the DGs (Zhou et al., 2019). The results are shown in the **Figure S6A**. Ct-infected cells had a large number of overlapping genes at three time points. PPI networks were constructed for these overlapping genes (**Figure S6B**), and most of them had functional interactions. **Figure S7C** showed the top 50 of central genes that play an important role in these overlapping genes, many of which were concentrated in function and may be generally regulated by other transcripts. The MCODE software in Cytoscape screened the subnet in PPI by clustering analysis (**Figure S7D**), which provides valuable information for the molecular mechanism of Ct-related diseases.

### Validation of Differentially Expressed Transcripts by RT-qPCR

**Figure 4:**
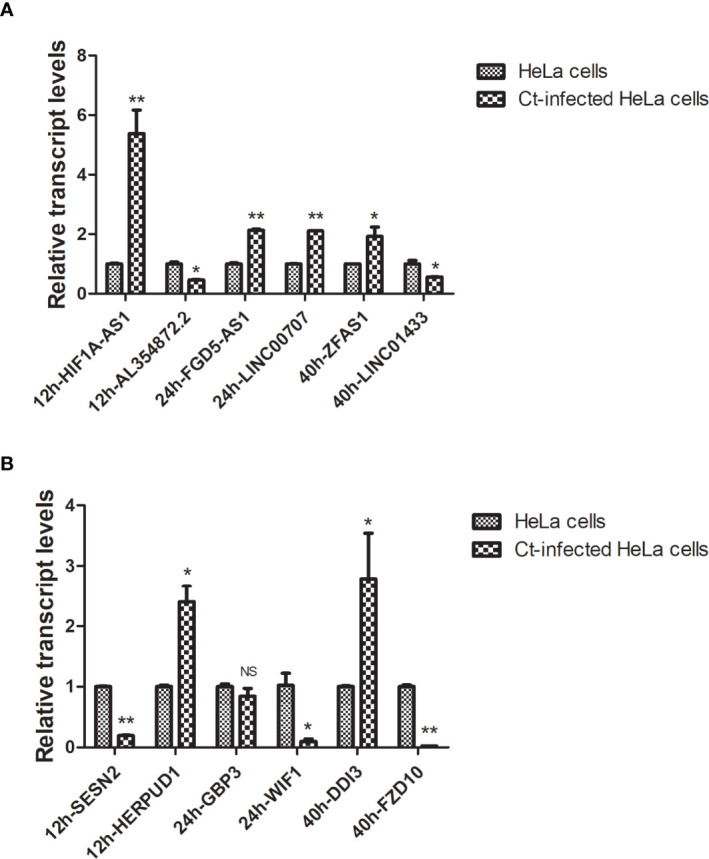
RT-qPCR validation of host differentially expressed lncRNAs and mRNAs in Ct-infected HeLa cells and uninfected cells. The relative transcriptional levels of six differentially expressed lncRNAs **(A)** and six differentially expressed mRNAs **(B)** at different post-infected hours in Ct-infected HeLa cells and uninfected cells were detected using RT-qPCR. The results were normalized with the endogenous *18S rRNA* control and measured the relative amount of target gene. Each sample was run in triplicate to ensure accurate fold change estimation, and the results were expressed as mean ± SD. *P<0.05; **P<0.01; NS, no significance.

To confirm the accuracy and repeatability of the microarray data, six of lncRNAs (HIF1A-AS1, AL354872.2, FGD5-AS1, LINC00707, ZFAS1, and LINC01433) in all samples were selected according to FC and literature. And six of mRNAs (SESN2, HERPUD1, GBP3, WIF1, FZD10, and DDIT3) were verified by RT-qPCR. The *18S rRNA* was used as an internal reference. The results showed that compared with the uninfected group, lncRNA HIF1A-AS1 expression was upregulated (*P*<0.01), and lncRNA AL354872.2 (*P*<0.05), mRNA molecule SESN2 (*P*<0.01), and HERPUD1 (*P*<0.05) expressions were downregulated at 12 hpi. LncRNA FGD5-AS1 (*P*<0.01) and LINC00707 (*P*<0.01) expressions were upregulated, and mRNA WIF1 expression was downregulated at 24 hpi (*P*<0.01). At 40 hpi, the expressions of lncRNA ZFAS1 (*P*<0.05) and mRNA DDIT3 (*P*<0.05) were upregulated, while that of lncRNA LINC01433 (*P*<0.05) and mRNA FZD10 (*P*<0.01) were downregulated (**Figure 4**). These DGs’ transcription levels were consistent with the results of the microarray.

### Ct Infection Resists Host Cell Apoptosis

**Figure 5:**
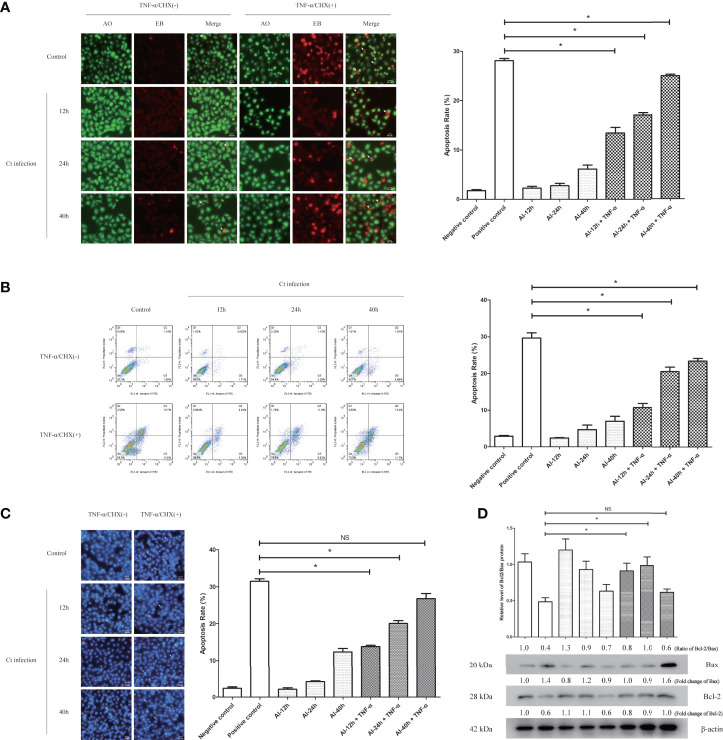
The anti-apoptotic effect of chlamydial infection. **(A)** AO/EB staining (10 × 20), **(B)** FCM, and **(C)** Hoechst staining (10 × 20) for a Ct-infected cells at different points with TNF-α treatment. Right panel is the apoptosis rate calculated by corresponding experiment. *P < 0.05. NS, no significance. **(D)** The expression of Bcl2 and Bax was measured by western blot in HeLa cells and Ct-infected cells after the apoptosis induction. The upper panel is the ratio of Bcl2/Bax, and calculated by the corresponding gray value *via* Quantity One. Arrows in **(A)** denote apoptotic cells, and in **(C)** indicate apoptotic bodies.

*Chlamydia* has been reported to resist host cell apoptosis and promote proliferation in the early stage and contribute to dissemination by inducing cell death in the late stage (Perfettini et al., 2003), which is one of the important mechanisms of *Chlamydia* for immune escape (Chen et al., 2019). In order to detect the anti-apoptosis effect of Ct infection, HeLa cells were infected with Ct for different time points and pretreated with 20 ng/ml TNF-α for 6 h, respectively. The results of AO/EB staining (**Figure 5A**) showed that compared with HeLa cells (27.36%), the apoptosis rates of Ct-infected cells at 12 and 24 h were 15.45 and 13.92% (*P* < 0.05), respectively, and that of Ct-infected cells at 40 h was 18.12% (*P* < 0.05). FCM results (**Figure 5B**) showed that compared with HeLa cells (29.63%), the apoptosis rates of cells at 12 and 24 hpi were 10.73 and 20.56% (*P* < 0.05), respectively, and that of 40 hpi was 23.34% (*P* < 0.05). As shown in the figure (**Figure 5C**), after inducing apoptosis, the apoptosis rates of cells at 12 and 24 hpi were 12.17 and 14.32%, both higher than uninfected cells (31.46%) (*P* < 0.01), and the apoptosis rate of cells infected with Ct for 40 h was 17.16% (*P* < 0.05). The Bcl-2/Bax ratio in control cells (0.486 ± 0.056) was lower than that of infected cells at 12 hpi (0.912 ± 0.107) and 24 hpi (0.988 ± 0.117), while having no significant difference at 40 hpi (0.617 ± 0.045) (**Figure 5D**). Ct-infected cells present anti-apoptotic effect at 12 and 24 hpi. With the extension of infection time, the anti-apoptotic ability of Ct decreased.

### Construction of Coding Non-Coding Co-Expression (CNC) Network

**Figure 6:**
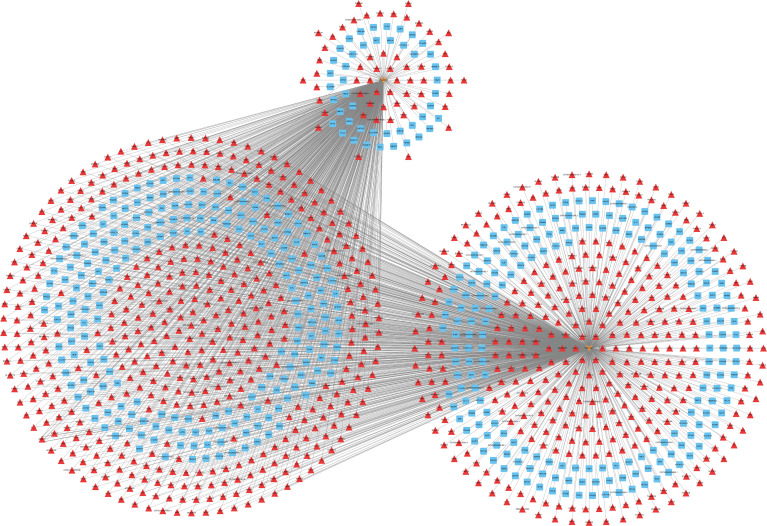
LncRNA-mRNA co-expression network in Ct-infected HeLa cells at 12 and 24 post-infected hours. The network was based on the Pearson relation coefficient of a lncRNA targeting an mRNA. Light orange shapes correspond to lncRNAs. Squares and triangle correspond to mRNAs. Red means upregulation, and blue means downregulation.

Ct needs to resist host cell apoptosis to achieve proliferation. Therefore, we further explored the anti-apoptosis mechanism of Ct. Among six of lncRNAs verified by RT-qPCR, HIF1A-AS1, FGD5-AS1, LINC00707, and LINC01433 were differentially expressed at 12 and 24 hpi. As HIF1A-AS1 and LINC01433 did not change the tendency at three time points of Ct infection (HIF1A-AS1 expression was upregulated at all three time points, while LINC01433 was downregulated), FGD5-AS1 and LINC00707 were chosen for further study. Co-expressed molecules of FGD5-AS1 and LINC00707 at 12 and 24 hpi were analyzed by calculation of PCC (PCC ≥ 0.7). Cytoscape 3.7.1 was used to create a CNC network. As shown in **Figure 6**, FGD5-AS1 was co-expressed with more differentially expressed molecules when compared to LINC00707. Co-Lnc software was used to analyze the pathway of FGD5-AS1 co-expressed molecules. The analysis results are shown in **Table 1**, among which Wnt signaling pathway is considered to be widely involved in the process of anti-apoptosis.

**Table 1:** Enrichment pathway of co-expression molecules of FGD5-AS1 at 12 and 24 hpi.

Accession	Name	# Genes	# CEGs	# Overlap	ePvalue	BH corr.
hsa03040	Spliceosome	128	394	8	0	0.005
hsa03030	Dna Replication	36	394	4	0	0.005
hsa04720	Long-Term Potentiation	70	394	5	0	0.008
hsa00563	Glycosylphosphatidylinositol Gpi Anchor Biosynthesis	25	394	3	0	0.008
hsa04114	Oocyte Meiosis	114	394	6	0.001	0.015
hsa04062	Chemokine Signaling Pathway	190	394	7	0.003	0.044
hsa04310	Wnt Signaling Pathway	151	394	6	0.003	0.044
hsa04672	Intestinal Immune Network for Iga Production	48	394	3	0.003	0.044
hsa04350	Tgf Beta Signaling Pathway	86	394	4	0.004	0.055
hsa04210	Apoptosis	88	394	4	0.005	0.055
hsa00590	Arachidonic Acid Metabolism	58	394	3	0.006	0.056
hsa03018	RNA Degradation	59	394	3	0.006	0.056
hsa04910	Insulin Signaling Pathway	137	394	5	0.008	0.056
hsa00830	Retinol Metabolism	64	394	3	0.008	0.056
hsa04620	Toll-Like Receptor Signaling Pathway	102	394	4	0.009	0.056
hsa04916	Melanogenesis	102	394	4	0.009	0.056
hsa04660	T Cell Receptor Signaling Pathway	108	394	4	0.012	0.064
hsa00982	Drug Metabolism Cytochrome P450	72	394	3	0.013	0.064
hsa05140	Leishmania Infection	72	394	3	0.013	0.064
hsa04662	B Cell Receptor Signaling Pathway	75	394	3	0.014	0.064
hsa04270	Vascular Smooth Muscle Contraction	115	394	4	0.015	0.064
hsa04010	Mapk Signaling Pathway	267	394	7	0.02	0.068
hsa04722	Neurotrophin Signaling Pathway	126	394	4	0.021	0.071
hsa04012	Erbb Signaling Pathway	87	394	3	0.024	0.077
hsa03010	Ribosome	88	394	3	0.025	0.077
hsa04020	Calcium Signaling Pathway	178	394	5	0.025	0.077
hsa04540	Gap Junction	90	394	3	0.026	0.08
hsa04144	Endocytosis	183	394	5	0.028	0.081
hsa04912	Gnrh Signaling Pathway	101	394	3	0.038	0.095

### Wnt/β-catenin Signaling Is Involved in the Anti-Apoptotic Effect of Ct-Infected Cells

**Figure 7:**
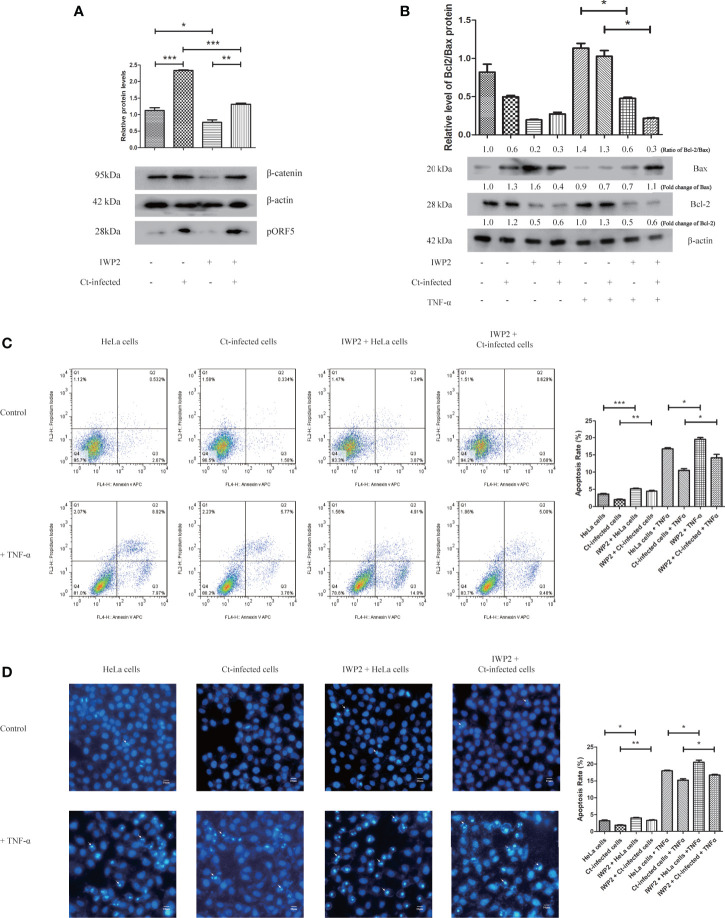
Inhibition of Wnt signaling pathway suppresses the anti-apoptotic effect of Ct-infected cells. **(A)** The protein expression level of β-catenin was measured by western blot in HeLa cells and Ct-infected cells after the addition of Wnt signaling pathway inhibitor IWP2 (10 μM). The upper panel is the corresponding gray value of β-catenin calculated by Quantity One. **(B)** The expression of Bcl2 and Bax was measured by western blot in HeLa cells and Ct-infected cells after the inhibition of Wnt signaling pathway. The upper panel is the ratio of Bcl2/Bax and calculated by the corresponding gray value *via* Quantity One. **(C, D)** The apoptotic rate of Ct-infected cells and uninfected cells was detected by FCM **(C)** and Hoechst staining **(D)** after addition of IWP2 (10 μM). The left panel is the apoptosis rate calculated by corresponding experiment. Arrows in **(C)** denote apoptotic bodies. *P < 0.05; **P < 0.01; ***P < 0.001.

Combined with the results in **Table 1**, we preliminarily explored whether Wnt/β-catenin signaling pathway was related to the anti-apoptosis effect of Ct. The results are shown in **Figure 7**. After inhibiting Wnt/β-catenin signaling pathway by inhibitor IWP2 (**Figure 7A**), the Bcl2/Bax ratio in the inhibited cells decreased (*P*<0.05) upon Ct infection (**Figure 7B**). The FCM results illustrated that the apoptosis rate (14.13%) in the Ct-infected inhibited cells was higher than that in the Ct-infected non-inhibited cells (10.47%) (*P*<0.05) after TNF-α induction (**Figure 7C**). Hoechst staining (**Figure 7D**) indicated that in the Ct-infected cells, the apoptosis rate of the inhibited cells (16.69%) was higher than that of the non-inhibited cells (14.15%) (*P*<0.05) after TNF-α induction. Wnt/β-catenin signaling pathway is involved in the anti-apoptotic effect of Ct.

### Upregulation of FGD5-AS1 Is Closely Associated With Apoptosis Resistance in Ct-Infected Cells

**Figure 8:**
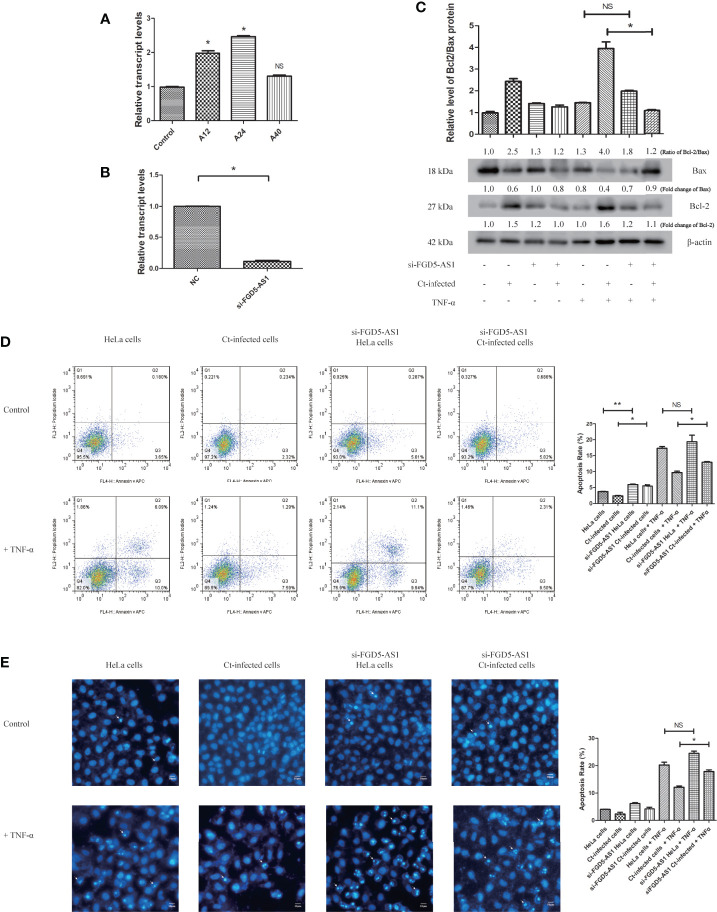
Interference of FGD5-AS1 inhibits the anti-apoptotic effect of Ct-infected cells. **(A)** RT-qPCR was used to detect the effect of acute infection on expression of FGD5-AS1 at 12 and 24 hpi. The *18S rRNA* was used as internal control. **(B)** RT-qPCR was used to detect the effect of FGD5-AS1 interference by siRNA; *18S rRNA* was used as internal control. **(C)** The expression of Bcl2 and Bax was measured by western blot in HeLa cells and Ct-infected cells after the interference of FGD5-AS1. The upper panel is the ratio of Bcl2/Bax and calculated by the corresponding gray value *via* Quantity One. **(D, E)** The apoptotic rate of Ct-infected cells and uninfected cells was detected by FCM **(D)** and Hoechst staining **(E)** after interference of FGD5-AS1. The right panel is the apoptosis rate calculated by corresponding experiment. Arrows in **(E)** denote apoptotic bodies. *P < 0.05; **P < 0.01; NS, no significance.

FGD5-AS1 participates in tumor invasion and migration through its anti-apoptotic effect (Fu et al., 2020; Wu et al., 2020; Li et al., 2021). Next, we investigated the effect of FGD5-AS1 on the anti-apoptotic effect of Ct. The results are shown in **Figure 8**. RT-qPCR detected the expression of FGD5-AS1 at 12 and 24 hpi, and the results were consistent with that in the microarray (**Figure 8A**). After inhibiting the expression of FGD5-AS1 (**Figure 8B**), the Bcl2/Bax ratio in the Ct-infected cells decreased compared with that in the non-interference group (*P*<0.05) (**Figure 8C**). The FCM results are shown in **Figure 8D**. After TNF-α induction, the apoptosis rate (15.05%) in the Ct-infected interfered cells was higher than that in the Ct-infected non-interfered cells (11.08%) (*P*<0.05). Hoechst staining (**Figure 8E**) showed that in the Ct-infected cells, the apoptosis rate of the interfered cells (17.70%) was higher than that of the non-interfered cells (11.12%) (*P*<0.05) after TNF-α induction. The above observations indicated that upregulation of FGD5-AS1 promoted apoptosis resistance during Ct infection.

### Ct Promotes the Activation of Wnt Signaling Pathway by Upregulating FGD5-AS1

**Figure 9:**
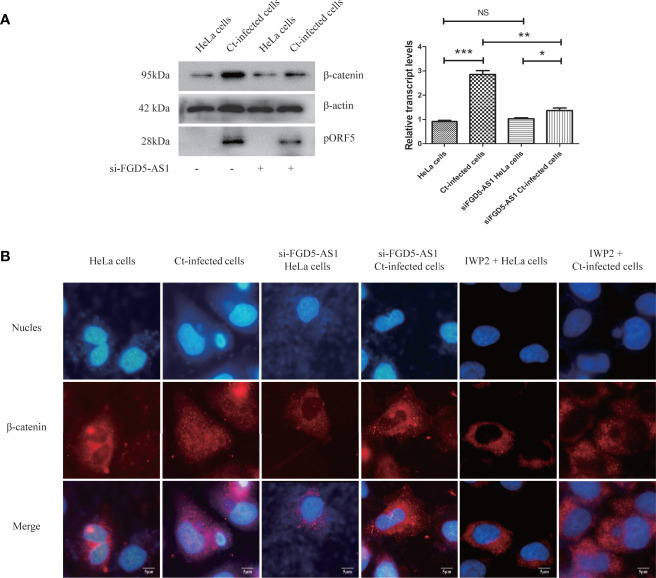
Interference of lncRNA FGD5-AS1 inhibits Wnt/β-catenin signaling pathway during Ct infection. **(A)** The protein expression level of β-catenin was measured by western blot in HeLa cells and Ct-infected cells after the interference of FGD5-AS1. The left panel is the corresponding gray value calculated by Quantity One. **(B)** The nuclear translocation of β-catenin in HeLa cells and Ct-infected cells after the interference of FGD5-AS1. *P < 0.05; **P < 0.01; ***P < 0.001; NS, no significance.

Follow-up experiments explored whether FGD5-AS1 is related to the regulation of Wnt/β-catenin signaling pathway in the anti-apoptotic process of Ct infection. Ct increased the expression of β-catenin (*P*<0.05) and promoted its nuclear translocation (**Figures 9A, B**). After interfering with FGD5-AS1, the expression of β-catenin during Ct infection was higher than that in the uninfected group (*P*<0.05), but significantly lower than that in the Ct-infected cells (*P*<0.01) (**Figure 9A**), and β-catenin nuclear translocation was suppressed (**Figure 9B**). These results proved that FGD5-AS1 was involved in the activation of Wnt/β-catenin signaling pathway during Ct infection.

**Figure 10:**
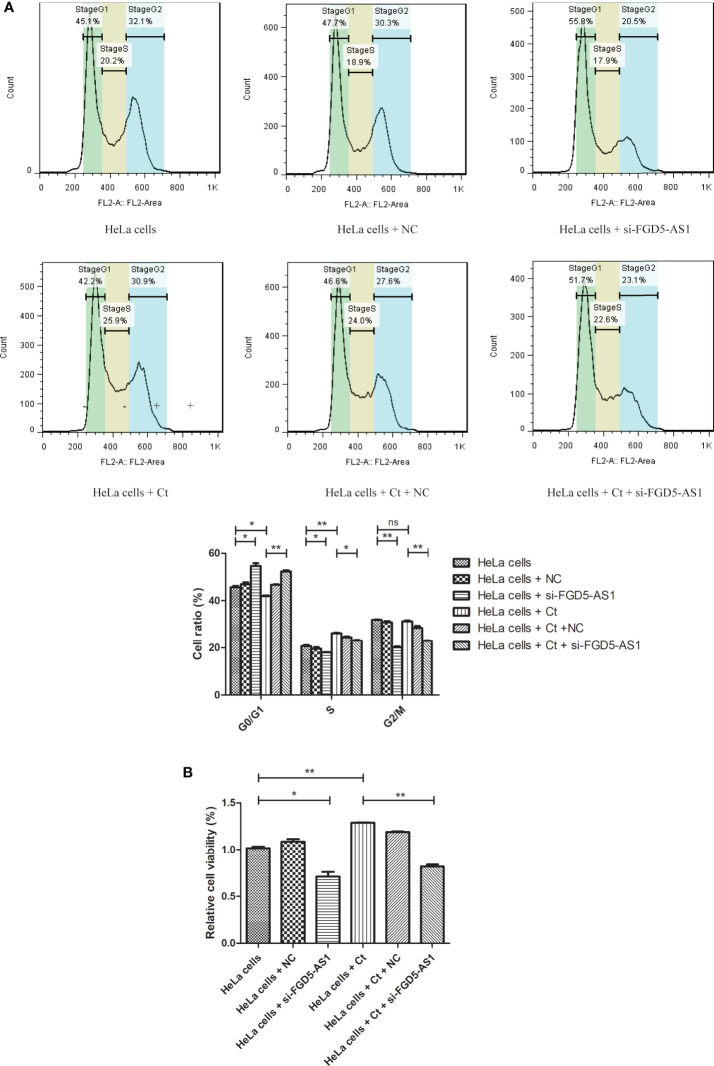
Interference of lncRNA FGD5-AS1 promotes the DNA replication at 12 hpi. **(A)** FCM analysis was used to detect the DNA content of control cells and FGD5-AS1-interfered cells. The lower panel is the corresponding cell percentage. **(B)** MTT was used to detect the cell viability of infected cells. *P < 0.05; **P < 0.01; NS, no significance.

It has been reported that Ct infection promotes host DNA damage and proliferation (Chumduri et al., 2013). As shown in **Figure S5A**, GO BP analysis showed that upregulated mRNAs were enriched in terms such as DNA replication at 12 hpi. The co-expressed molecules of FGD5-AS1 were the same as involved terms of differentially expressed mRNAs at 12 hpi (**Table 1**). To evaluate the effect of FGD5-AS1 on DNA replication and cell cycle, FCM analysis was used to detect the DNA content of FGD5-AS1-interfered cells. The results are shown in **Figure 10**. The percentages of cells in various cell cycle stages were different from control cells, and the interference of FGD5-AS1 and Ct infection aroused cell cycle change. After interference with the expression of FGD5-AS1, the percentage of cells in G1/G0 phase increased (45.1 *vs* 55.8%) (*P*<0.05), and that in S phase (20.2 *vs* 17.9%) (*P*<0.05) and G2/M (32.1 *vs* 20.5%) (*P*<0.01) decreased. Ct infection increased the percentage of cells in S phase (20.2 *vs* 25.9%) (*P*<0.01) and was accompanied by a decrease in G1/G0 phase (45.1 *vs* 42.2%) (*P*<0.05). Interestingly, FGD5-AS1-interfered cells presented a decrease in the percentage of cells in the G2/M phase (*P*<0.01), but Ct infection did not affect the percentage of cells in the G2/M phase (32.1 *vs* 30.9%) (*P*>0.05), indicating that FGD5-AS1 participated in the promotion of DNA replication during Ct infection. The MTT test detected the cell viability of infected cells. The results showed that cell viability increased (*P*<0.05) after Ct infection. Regardless of whether Ct was infected or not, interference with FGD5-AS1 reduced the cell viability of HeLa cells (*P*<0.01). The above results indicate that Ct infection can increase DNA replication and cell viability *via* FGD5-AS1 upregulation.

## Discussion

Due to the characteristics of intracellular survival, Ct relies on host cells for replication, proliferation, and differentiation. Therefore, Ct needs to manipulate the host cell physiological process to complete growth and development. Recent studies have shown that a class of ncRNA called circular RNA (circRNA) in Ct-infected cells was dysregulated and participated in the interaction between Ct and host cells (Liu et al., 2019). Another type of ncRNAs, lncRNA, as an important class of gene expression regulatory elements, is a key regulator of genes in different physiological and pathological processes (Zhao et al., 2020). In recent years, the role of lncRNAs in pathogenic microbial infections has gradually attracted people’s attention. However, it is not clear whether lncRNA is involved in the pathogenesis of *Chlamydia*. We selected 12, 24, and 40 hpi as the representative time points during Ct infection and detected the differentially expressed lncRNAs at different time points. We determined that 165 of lncRNAs and 459 of mRNAs have been continuously upregulated, while 80 of lncRNAs and 37 of mRNAs have been continuously downregulated after Ct infection. By inhibiting the expression of candidate lncRNA FGD5-AS1, it was proved that it participates in the DNA replication and anti-apoptosis process during Ct infection. This provides new insights for the research that Ct regulates the host lncRNAs.

LncRNA and mRNA expression profiles have been widely used to explore the molecular mechanisms of disease pathogenesis (such as cancers, viral infections, and bacterial infections) (Bhan et al., 2017; Chen et al., 2018; Fathizadeh et al., 2020). The research of lncRNAs primarily focuses on facultative intracellular bacteria in bacterial infections. For instance, Mtb increased the expression of lnc-CD244, thereby recruiting EZH2 to mediate the trimethylation of H3K27 on IFN-γ/TNF-α locus, suppressing the expression of IFN-γ and TNF-α and evading the host immune response (Wang et al., 2015). Hp upregulated the expression of RING1/RAD51 by upregulating lncRNA SNHG17 as a ceRNA for miR-3909, changed the DNA repair system, and promoted the occurrence of gastric cancer. Therefore, as an intracellular pathogen, Ct may also be involved in the regulation of host signaling pathways such as apoptosis and autophagy by alternating the host lncRNA expression profile, so as to affect the survival and reproduction of Ct in host cells. Here, we confirmed that the differentially expressed lncRNAs in Ct-infected HeLa cells at 12, 24, and 40 hpi were 2,130, 1,081, and 1,101, respectively, demonstrating that Ct infection led to differential expression of host lncRNAs, and the host lncRNAs were widely changed at 12 hpi. Among these candidate lncRNAs, we randomly screened and verified the expressions of FGD5-AS1 and LINC00707. Both of them were upregulated at 12 and 24 hpi, but there was no significant difference in expression at 40 hpi, suggesting that these two lncRNAs may be involved in the biological process induced by Ct in these two time points.

The results of CNC analysis showed that the co-expression differential mRNAs of FGD5-AS1 were enriched in multiple signaling pathways including DNA replication and Wnt/β-catenin signaling pathway. Our follow-up studies confirmed that the inhibition of FGD5-AS1 affected the nuclear translocation of β-catenin. FGD5-AS1 has been reported to regulate signaling pathways by regulating different molecules in different cancer cells such as colorectal cancer cells, esophageal squamous cell carcinoma cells, and non-small-cell lung cancer cells, so as to promote the proliferation and migration of these cancer cells (Li et al., 2019; Fan et al., 2020; Gao et al., 2020). It is indicating that FGD5-AS1 plays an oncogene in different cell lines. Wu et al. reported that FGD5-AS1 activated Wnt signaling pathway by regulating miR-129-5p/HNRNPK axis and promoted glioblastoma progression (Wu et al., 2020). Chlamydia can cooperate with HPV to induce cervical cancer and activate Wnt signaling pathway in Ishikawa and Hec-1b cell lines, as well as fallopian tube tissue, suggesting that Ct upregulates FGD5-AS1 to inhibit apoptosis *via* Wnt signaling pathway, which may be the general mechanism of Ct pathogenesis (Kessler et al., 2012; Kintner et al., 2017). However, whether the effect of FGD5-AS1 on the Wnt/β-catenin signaling pathway is directly binding to mRNAs or competitive binding to certain miRNAs for gene expression regulation remains to be further studied.

We found that the expression patterns of lncRNAs and mRNAs were significantly different at 12 hpi, and there were many same DGs at 12 and 24 hpi. GO and KEGG pathway analysis helped us to further predict the potential function of differentially expressed mRNAs. The upregulated mRNAs at various stages were enriched in “Spliceosome,” “RNA transport,” “Ribosome biogenesis in eukaryotes,” confirming that the intracellular organism Ct needs to obtain nutrient from the host. In accordance with the intracellular survival characteristics, the upregulated DGs at various time points were also generally enriched in “NOD-like receptor signaling pathway,” “RIG-I-like receptor signaling pathway,” “Epstein-Barr virus infection,” etc. Terms related to intracellular recognition of receptors indicated that Ct could activate the host cell’s intracellular receptors during its intracellular survival. We also found that the downregulated mRNAs at all time points were enriched in the calcium pathway. During the Ct infection, the calcium in the cells was redistributed, resulting in local accumulation of calcium ions near Ct inclusion. We speculate that due to the decrease in cytosolic calcium ion concentration, the downstream calcium signaling pathway is inhibited (Majeed et al., 1999). Compared with 12 and 24 hpi, 40 hpi had more unique differentially expressed lncRNAs. This observation is consistent with the phenomenon that Ct evades host immunity in the early and middle stages for intracellular proliferation and induces cell death in the late stage to spread. For further research, we constructed a CNC network and predicted the enrichment pathway of co-expressed molecules. In this case, the enrichment analysis of FGD5-AS1 co-expressed mRNAs in 12 hpi revealed that these mRNAs were mainly enriched in the Wnt/β-catenin signaling pathway. Wnt signaling pathway plays a vital role in anti-apoptosis, primarily including classic Wnt pathway (Wnt/β-catenin pathway), Wnt/PCP (planar cell polarity) pathway, and Wnt/calcium ion (Wnt/Ca^2+^). Wnt/PCP plays a role by activating downstream to activate JNK, while Wnt/Ca^2+^ can activate protein kinase C (PKC) in the cAMP pathway to cause an increase in intracellular calcium ion concentration, and can antagonize the classic Wnt signaling pathway (MacDonald et al., 2009; De, 2011; VanderVorst et al., 2019). However, both the cAMP signaling pathway and the calcium signaling pathway were downregulated at 12 and 24 hpi (**Figure S5**), suggesting that Ct activated the classic Wnt/β-catenin signaling pathway but not the Wnt/Ca^2+^ signaling pathway, which was consistent with our results.

In addition, Ct infection is related to the occurrence of cervical cancer and ovarian cancer, but the exact mechanism has not been clarified. Previous studies have shown that Ct infection can increase the phosphorylation level of H2AX and H2k9me3 in cervical epithelial cells, which were the hallmarks of DNA double-strand breaks (DSBs) and senescence-associated heterochromatin foci (SAHF), respectively (Chumduri et al., 2013). Although DSBs were accompanied by apoptosis, the Ct-infected cells still proliferated because of SAHF and were prone to malignant transformation due to their unstable genome. This provides experimental evidence for the relationship between Ct infection and female reproductive system cancer. Consistent with the previous study, we found that upregulated mRNAs at 12 hpi were enriched in DNA replication according to KEGG analysis. And the DNA replication level of host cells was increased at 12 hpi. LncRNAs have been shown to regulate DNA methylation and histone modification to change chromatin status, thereby affecting transcription during bacterial infection (Wen et al., 2020a). *Mycobacterium smegmatis* upregulated the expression of MEG3 to inhibit promoter hypermethylation, repressing the expression of TGF-β by forming RNA-DNA triplex structure (Sharbati et al., 2019). Similar to *Mycobacterium*, Ct is an intracellular microbial pathogen. Whether Ct can change chromatin status by regulating lncRNAs to participate in immune escape and malignant transformation is one of the most potential research directions of interaction between Ct and host in the future.

Effective diagnosis of Ct infection is essential for the treatment and the control of Ct-related STI. In recent years, more and more evidence showed that lncRNA can be used as a molecular biomarker for disease diagnosis and post-diagnosis. For example, circulating lncRNA-HULC may be a candidate serum tumor marker for early diagnosis of gastric cancer and monitoring its progress and prognosis (Gao et al., 2015). Oncogenic HPV can promote the expression of lnc-GANCI-2, which may be one of the infection markers of high-risk HPV (Liu et al., 2021). However, there is no report on the utilization of lncRNAs as biomarkers for Ct infection. In our study, there are many significantly differentially expressed lncRNAs induced by Ct, which suggests that lncRNAs may become new biomarkers of Ct infection. The next research direction of our team will focus on the application of lncRNAs as potential diagnostic markers for Ct infection in the clinic.

In conclusion, our research showed that Ct extensively influences the expressions of lncRNAs and mRNAs during infection. DNA replication and apoptosis of host cells were affected by upregulating FGD5-AS1 *via* Wnt/β-catenin pathway during Ct infection. Screening these differentially expressed lncRNAs may help to clarify the pathogenic mechanism of Ct infection, but the key underlying molecular mechanism of the Ct-host interaction still needs deeper study. Further mechanism research of these multifunctional molecules is essential, which will broaden our understanding of the pathogenesis of Ct and provide new methods for the diagnosis and treatment of Ct infection.

## Data Availability Statement

The datasets presented in this study can be found in online repositories. The names of the repository/repositories and accession number(s) can be found below: https://www.ncbi.nlm.nih.gov/geo/, GSE165628.

## Author Contributions

ZL, YW, and FL: Conceived and designed the experiments. LZ, SS, and WL: Contributed to experimental technique support. YL and KS: Provided much scientific advice for this study. ZL, YW, and FL analyzed the data. YW wrote the manuscript. All authors contributed to the article and approved the submitted version.

## Funding

This work was supported by the National Natural Science Foundation of China (No. 32070189, 81772210, and 31900160), the Key Program of Hunan Provincial Department of Education (No. 20A421), Clinical Research Project of University of South of China (No. USCKF201902K01).

## Conflict of Interest

The authors declare that the research was conducted in the absence of any commercial or financial relationships that could be construed as a potential conflict of interest.

## Publisher’s Note

All claims expressed in this article are solely those of the authors and do not necessarily represent those of their affiliated organizations, or those of the publisher, the editors and the reviewers. Any product that may be evaluated in this article, or claim that may be made by its manufacturer, is not guaranteed or endorsed by the publisher.

## Supplementary Material

Supplementary Figure 1 Hierarchical clustering analysis of gene expression levels between Ct-infected HeLa cells and uninfected HeLa cells. LncRNAs **(A)** and mRNAs **(B)** were significantly changed in response to Ct infection. The clustering analysis was performed by MeV 4.9.0 software. Red and green indicate upregulated and downregulated DE genes, respectively.

Supplementary Figure 2 Volcano plots of DGs in Ct-infected HeLa cells and HeLa cells at different post-infected hours. Volcano plots exhibit the differentially expressed lncRNAs **(A)** and mRNAs **(D)** at 12 hpi, the differentially expressed lncRNAs **(B)** and mRNAs **(E)** at 24 hpi, and the differentially expressed lncRNAs **(C)** and mRNAs **(F)** at 40 hpi. Red and green indicate upregulated and downregulated DE genes, respectively.

Supplementary Figure 3 Scatter plots of DGs in Ct-infected HeLa cells and HeLa cells at different post-infected hours. Scatter plots exhibit the differentially expressed lncRNAs **(A)** and mRNAs **(D)** at 12 hpi, the differentially expressed lncRNAs (**B**) and mRNAs **(E)** at 24 hpi, and the differentially expressed lncRNAs **(C)** and mRNAs **(F)** at 40 hpi. Red and green indicate upregulated and downregulated DGs, respectively.

Supplementary Figure 4 Gene ontology analysis of DGs in Ct-infected HeLa cells and HeLa cells at different post-infected hours. The bar plot shows Fold Enrichment value of the significant enrichment terms and pathway analysis for upregulated mRNAs at 12 **(A)**, 24 **(C)**, and 40 **(E)** post-infected hours, and for downregulated mRNAs at 12 **(B)**, 24 **(D)**, and 40 **(F)** post-infected hours. Red bars indicate biological process, green bars represent cellular components, and blue bars means molecular function.

Supplementary Figure 5 KEGG pathway analysis of DGs in Ct-infected HeLa cells and HeLa cells at different post-infected hours. The bar plot shows KEGG pathway analysis for upregulated mRNAs at 12 **(A)**, 24 **(C)**, and 40 **(E)** post-infected hours, and for downregulated mRNAs at 12 **(B)**, 24 **(D)**, and 40 **(F)** post-infected hours.

Supplementary Figure 6 Protein-protein interaction (PPI) network of differentially expressed mRNAs. **(A)** Circos diagram shows the differentially expressed mRNAs at three time points. Red indicates all differential genes at 12 hpi, blue indicates all differential genes at 24 hpi, and green indicates all differential genes at 40 hpi. The light orange in the inner circle indicates the unique differential genes at that time point, the dark orange indicates the overlap of genes at different time points, the purple line indicates the overlap between genes, and the blue line indicates the related function between genes. **(B)** Differentially expressed mRNAs at three time points are used to construct a PPI network by Metascape software, and visualized by Cytoscape 3.7.1. **(C)** The top 50 hub mRNAs from PPI were included using Edgecount. The closer to red, the more central the gene is. The size of the circle represents the significance of a gene. **(D)** Sub-networks screened from PPI network by MCODE in Cytoscape. A kind of colors represents a cluster participate in the same biological process.
