# Supplementary figures and images for: Long Non-Coding RNA FGD5-AS1 Induced by Chlamydia trachomatis Infection Inhibits Apoptosis via Wnt/β-Catenin Signaling Pathway
Source: Front Cell Infect Microbiol. 2021 Sep 9;11:701352. doi: 10.3389/fcimb.2021.701352 (PMC8460124; doi:10.3389/fcimb.2021.701352)

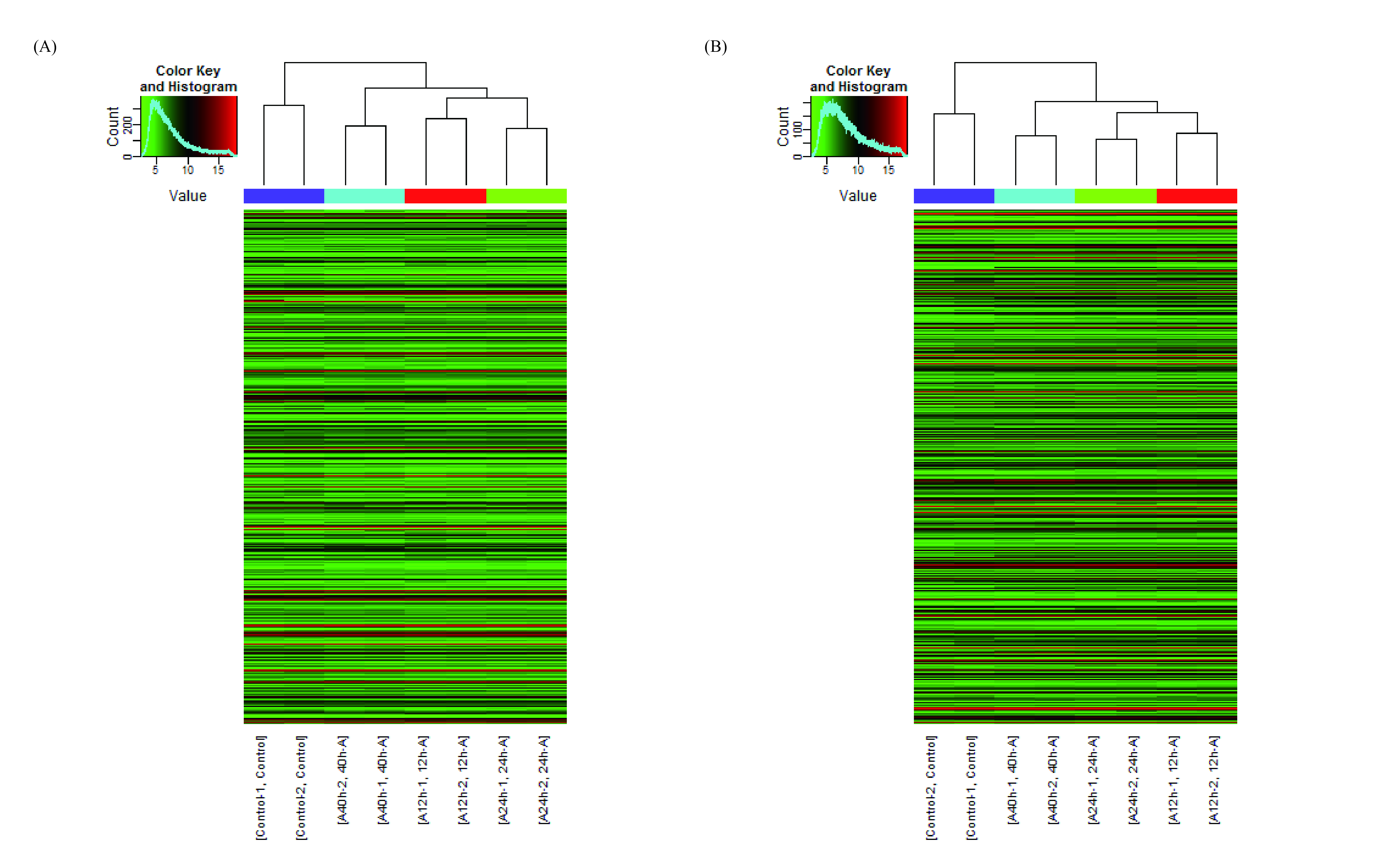

Supplement: Supplementary Figure 1 — Hierarchical clustering analysis of gene expression levels between Ct-infected HeLa cells and uninfected HeLa cells. LncRNAs (A) and mRNAs (B) were significantly changed in response to Ct infection. The clustering analysis was performed by MeV 4.9.0 software. Red and green indicate upregulated and downregulated DE genes, respectively. [file Image_1.tif]

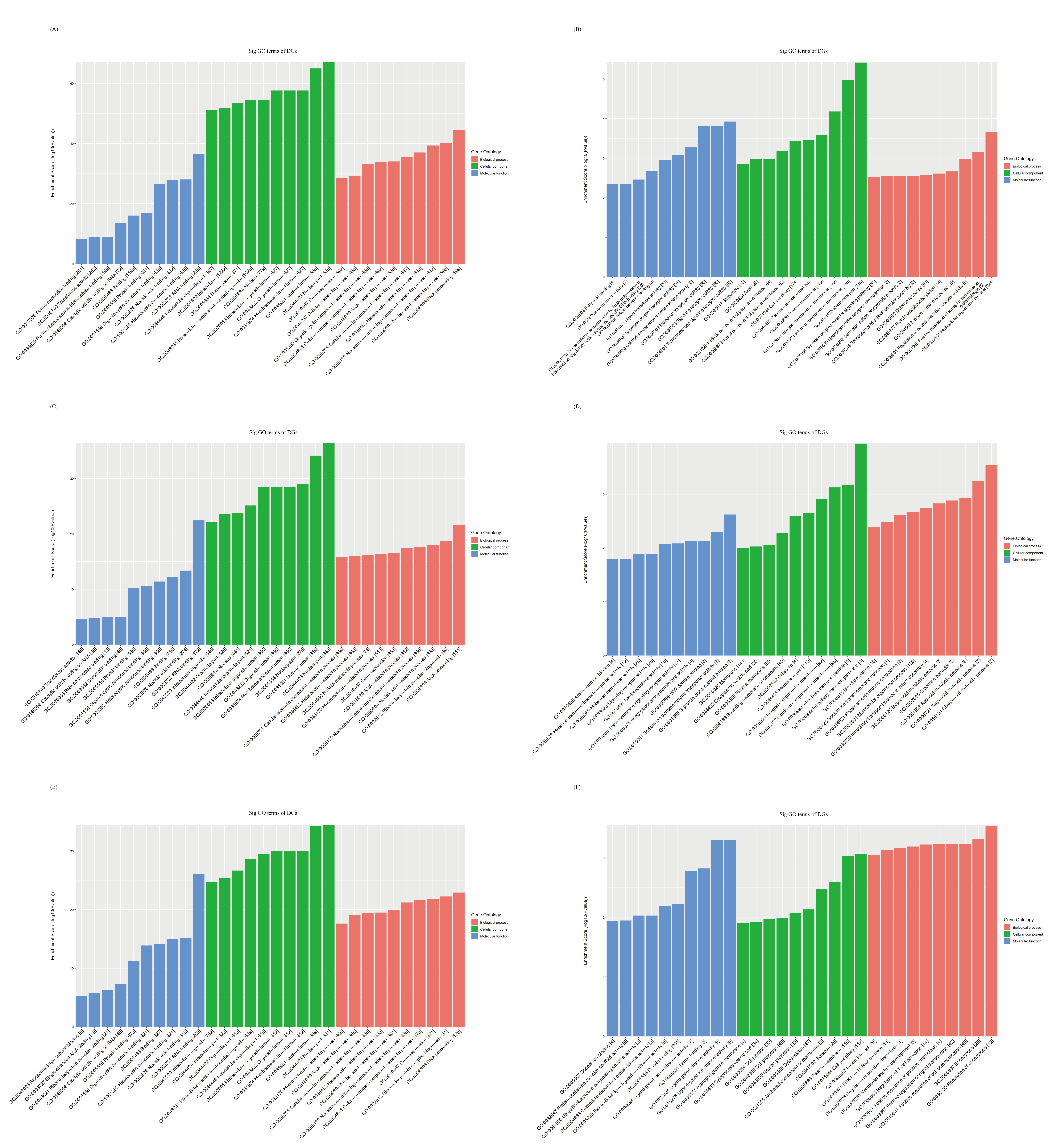

Supplement: Supplementary Figure 4 — Gene ontology analysis of DGs in Ct-infected HeLa cells and HeLa cells at different post-infected hours. The bar plot shows Fold Enrichment value of the significant enrichment terms and pathway analysis for upregulated mRNAs at 12 (A), 24 (C), and 40 (E) post-infected hours, and for downregulated mRNAs at 12 (B), 24 (D), and 40 (F) post-infected hours. Red bars indicate biological process, green bars represent cellular components, and blue bars means molecular function. [file Image_4.tif]

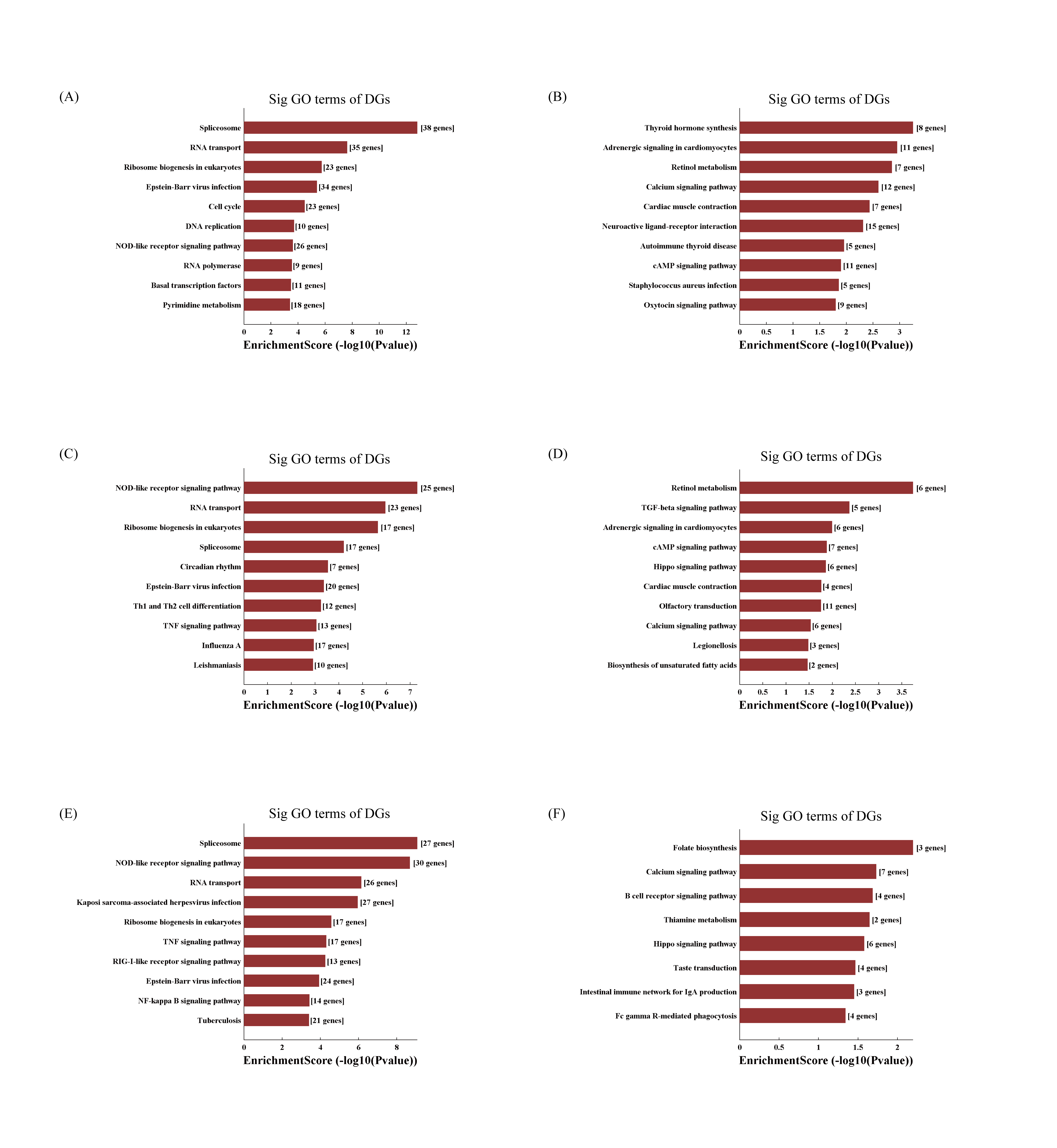

Supplement: Supplementary Figure 5 — KEGG pathway analysis of DGs in Ct-infected HeLa cells and HeLa cells at different post-infected hours. The bar plot shows KEGG pathway analysis for upregulated mRNAs at 12 (A), 24 (C), and 40 (E) post-infected hours, and for downregulated mRNAs at 12 (B), 24 (D), and 40 (F) post-infected hours. [file Image_5.tif]

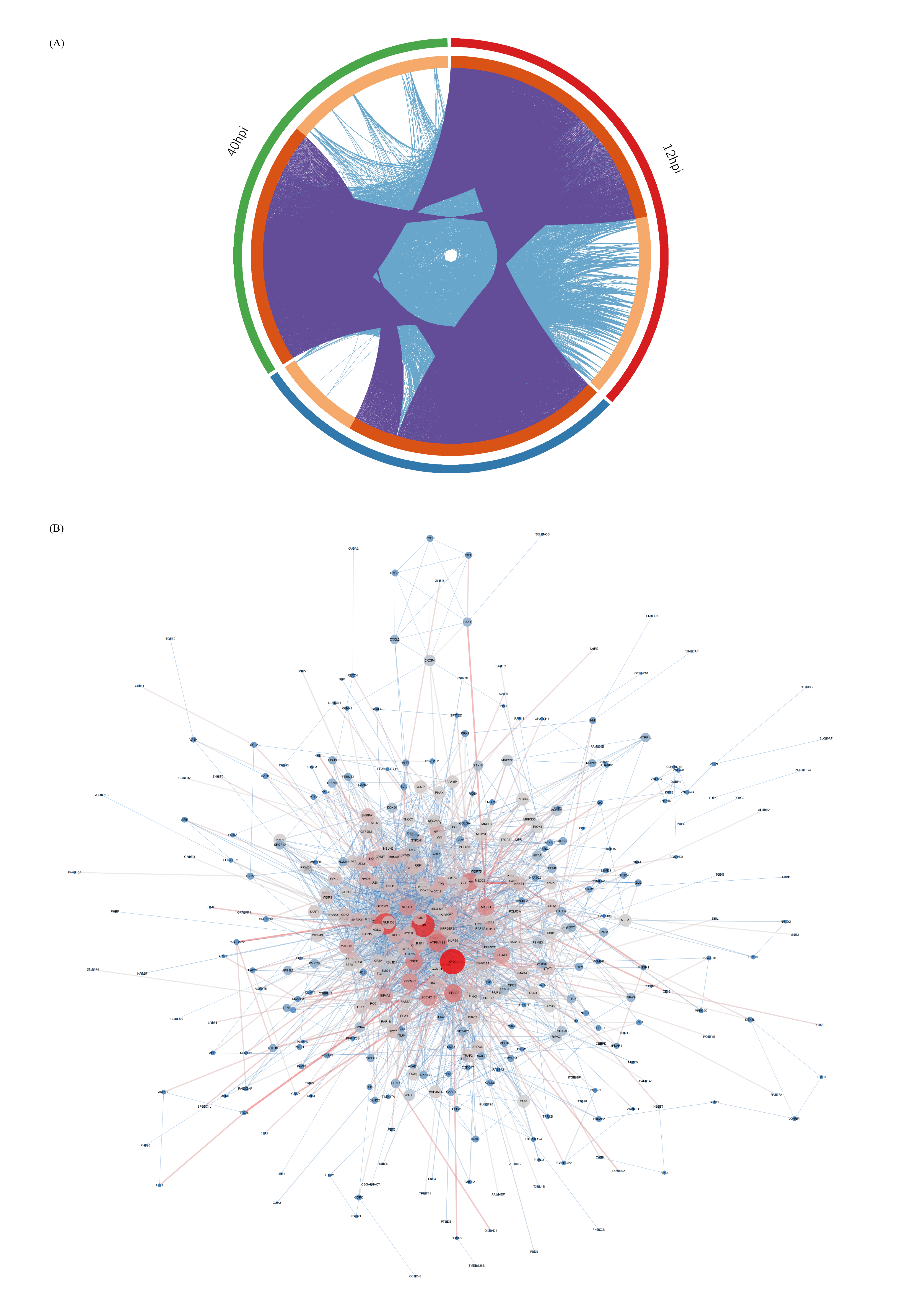

Supplement: Supplementary Figure 6 — Protein-protein interaction (PPI) network of differentially expressed mRNAs. (A) Circos diagram shows the differentially expressed mRNAs at three time points. Red indicates all differential genes at 12 hpi, blue indicates all differential genes at 24 hpi, and green indicates all differential genes at 40 hpi. The light orange in the inner circle indicates the unique differential genes at that time point, the dark orange indicates the overlap of genes at different time points, the purple line indicates the overlap between genes, and the blue line indicates the related function between genes. (B) Differentially expressed mRNAs at three time points are used to construct a PPI network by Metascape software, and visualized by Cytoscape 3.7.1. (C) The top 50 hub mRNAs from PPI were included using Edgecount. The closer to red, the more central the gene is. The size of the circle represents the significance of a gene. (D) Sub-networks screened from PPI network by MCODE in Cytoscape. A kind of colors represents a cluster participate in the same biological process. [file Image_6.tif]

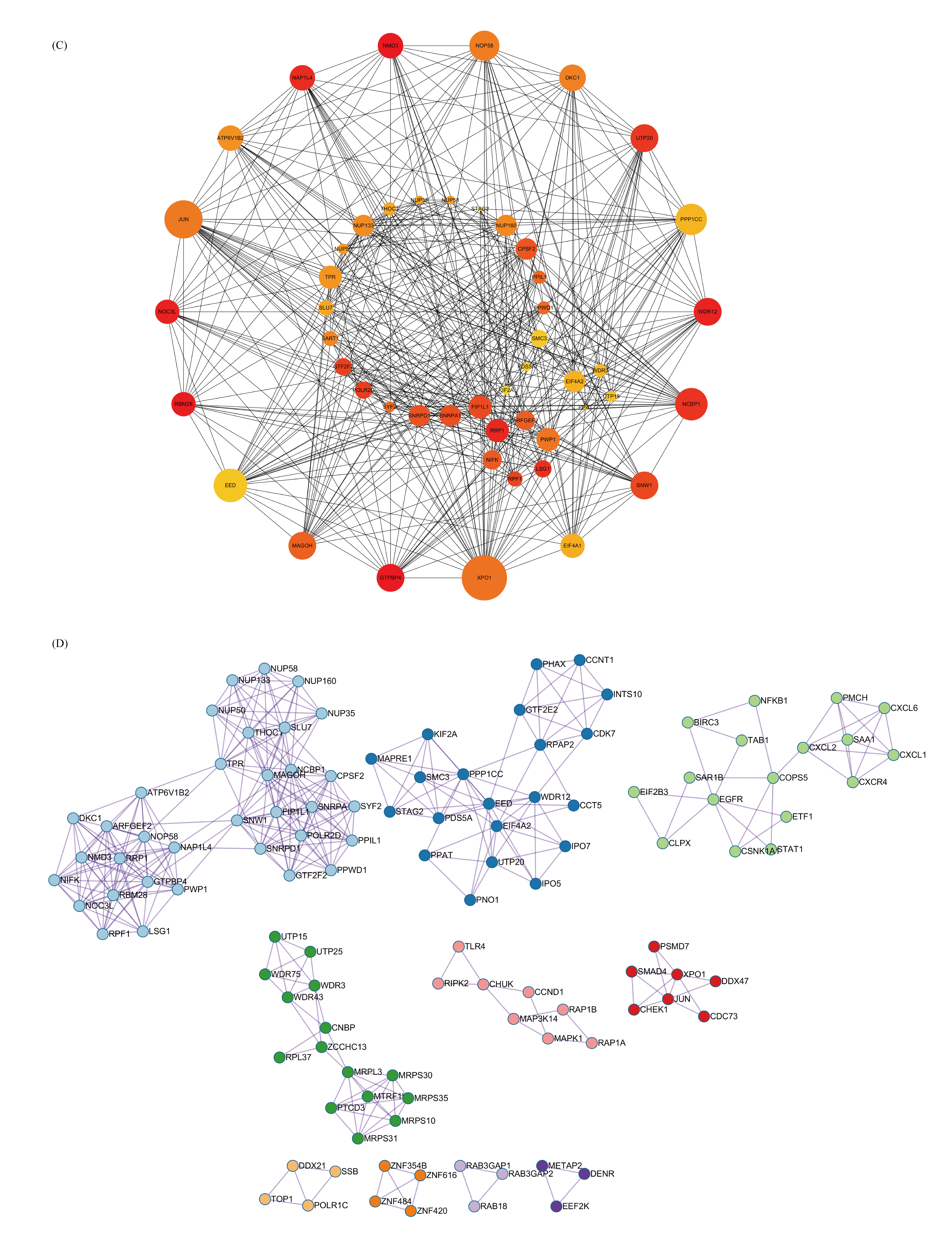

Supplement: Supplementary file 7 [file Image_7.tif]
